# β2-Chimaerin, a GTPase-Activating Protein for Rac1, Is a Novel Regulator of Hepatic Insulin Signaling and Glucose Metabolism

**DOI:** 10.3390/molecules29225301

**Published:** 2024-11-09

**Authors:** Cristian Andrés Carmona-Carmona, Pablo Zini, Eladio A. Velasco-Sampedro, Irene Cózar-Castellano, Germán Perdomo, María J. Caloca

**Affiliations:** 1 Instituto de Biomedicina y Genética Molecular (IBGM), CSIC-UVA, 47003 Valladolid, Spain; cristianandres.carmonacarmona@univr.it (C.A.C.-C.); zinipablo@fceqyn.unam.edu.ar (P.Z.); eavelsam@uva.es (E.A.V.-S.); irene.cozar@uva.es (I.C.-C.); g.perdomo@csic.es (G.P.); 2 Centro de Investigación Biomédica en Red de Diabetes y Enfermedades Metabólicas Asociadas (CIBERDEM), 28029 Madrid, Spain

**Keywords:** β2-chimaerin, Rac1, GTPase activating protein (GAP), AKT, insulin signaling, glucose metabolism, liver

## Abstract

Glucose homeostasis is a complex process regulated by multiple organs and hormones, with insulin playing a central role. Recent evidence underscores the role of small GTP-binding proteins, particularly Rac1, in regulating insulin secretion and glucose uptake. However, the role of Rac1-regulatory proteins in these processes remains largely unexplored. In this study, we investigated the role of β2-chimaerin, a Rac1-specific GTPase-activating protein (GAP), in glucose homeostasis using whole-body β2-chimaerin knockout mice. Our data revealed that β2-chimaerin deficiency results in improved glucose tolerance and enhanced insulin sensitivity in mice. These metabolic effects were associated with increased insulin-induced AKT phosphorylation in the liver and activation of downstream pathways that regulate gluconeogenesis and glycogen synthesis. We show that insulin activates Rac1 in the liver. However, β2-chimaerin deletion did not significantly alter Rac1 activation in this organ, suggesting that β2-chimaerin regulates insulin signaling via a Rac1-independent mechanism. These findings expand our understanding of Rac1 regulation in glucose metabolism, and identify β2-chimaerin as a novel modulator of hepatic insulin signaling, with potential implications for the development of insulin resistance and diabetes.

## 1. Introduction

Glucose homeostasis is a tightly regulated metabolic process that maintains blood glucose levels within a very narrow range in healthy individuals. This process involves various physiological mechanisms and multiple tissues and organs, including the pancreas, liver, muscle, and adipose tissue, and it is regulated by the actions of several hormones and extracellular signals, with insulin playing a pivotal role [1].

Recently, the Rho GTPase family of small G-proteins has emerged as critical regulators of glucose homeostasis. Among these proteins, Rac1 plays a prominent role in controlling glucose-stimulated insulin secretion and insulin-stimulated glucose uptake [2,3]. The main cellular function of Rac1 is the control of actin cytoskeleton dynamics, a process highly relevant for metabolic regulation. In pancreatic beta cells, the translocation of insulin-containing vesicles to the plasma membrane is mediated through actin cytoskeleton rearrangements that depend on Rac1 activation [4,5]. Similarly, in skeletal muscle and adipose tissue, insulin-induced GLUT4 translocation is linked to cytoskeletal dynamics [6,7,8]. Rac1 has a demonstrated role in this process in muscle cells, with in vitro and in vivo studies showing that Rac1 activation by insulin induces the reorganization of the cortical actin cytoskeleton that leads to GLUT4 translocation and the concomitant insulin-stimulated glucose uptake [9,10,11]. In adipose tissue, other Rho GTPases seem to have a more prominent role in insulin-induced glucose uptake [11,12,13], although recent findings indicate that Rac1 also participates in GLUT4 translocation in insulin-stimulated adipocytes [14]. According to this crucial role of Rac1 in maintaining glucose homeostasis, dysregulated Rac1 signaling is associated with metabolic disorders. Hyperactivation of Rac1 is observed in islets from patients and mouse models of type 2 diabetes [15,16], whereas in skeletal muscle, Rac1 signaling is diminished in insulin-resistant states in both mice and humans [9,17].

Despite the importance of proper Rac1 activation for maintaining whole-body metabolic homeostasis, the molecular mechanisms governing Rac1 activity in metabolic contexts are not yet fully elucidated. Rac1 functions as a molecular switch, cycling between an inactive GDP-bound form and an active GTP-bound form [18]. This process is tightly regulated by three types of proteins: Guanine nucleotide Exchange Factors (GEFs), GTPase-activating proteins (GAPs), and Guanine Dissociation Inhibitors (GDIs). GEFs activate Rac1 while GAPs and RhoGDIs inhibit Rac1 signaling [19,20]. Among the Rac1-GEFs, members of the P-Rex family (P-Rex1 and 2), the Vav family (Vav2 and Vav3), Tiam1, β-PIX Plekhg4, and Kalirin play roles in regulating glucose-stimulated insulin release in pancreatic β-cells and/or insulin-stimulated glucose uptake in skeletal muscle or adipose tissue [21]. Notably, these GEFs are expressed in the liver [21], but whether Rac1 has glucoregulatory roles in this organ remains unexplored. Consequently, the exact function of GEFs in hepatic metabolism is largely unknown.

The role of the negative regulators of Rac1 is limited to a recent report of RhoGDIα as an important modulator of skeletal muscle insulin sensitivity [22]. The specific GAP proteins involved in controlling Rac1 activity in these processes remain unidentified. A potential candidate is β2-chimaerin, as genetic studies have linked this GAP protein with diabetes. β2-chimaerin is one of the members of the chimaerin family which are highly selective for Rac1 [23]. Haploinsufficiency of the gene encoding for β2-chimaerin (*CHN2*) in combination with the insulin receptor gene (INSR) was detected in a family with insulin resistance and early diabetes onset, being one of the few examples of the digenic cause of disease in diabetic patients [24]. This result first implicated β2-chimaerin in insulin signaling in vivo. Further supporting the role of β2-chimaerin in glucose metabolism, polymorphisms in the *CHN2* gene have been associated with the development of diabetes and its complications [25,26]. Although current data are limited, these findings underscore the necessity for further research to delineate the contribution of β2-chimaerin to the regulation of glucose homeostasis. In this study, we used whole-body β2-chimaerin knockout mice to investigate this important issue and, for the first time, demonstrated that β2-chimaerin influences insulin signaling in the liver.

## 2. Results

### 2.1. β2-Chimaerin Deficiency Leads to Improved Glucose Tolerance

**Figure 1:**
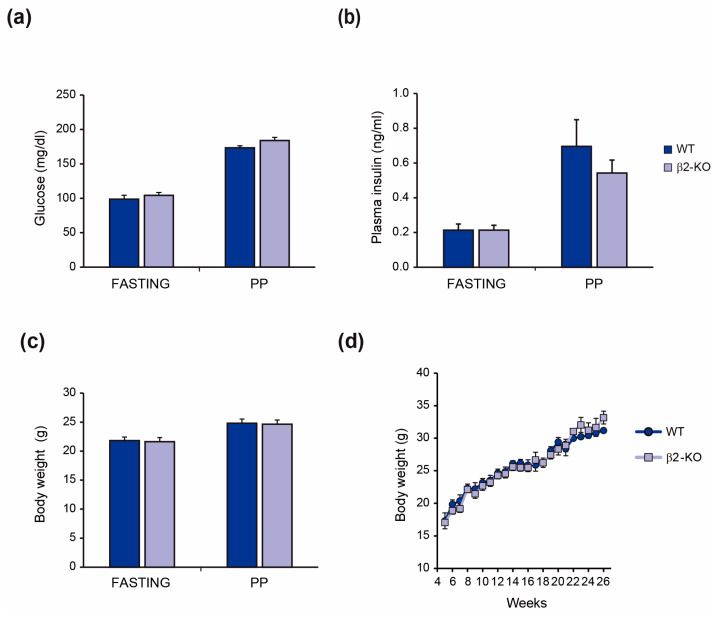
Metabolic features of β2-chimaerin KO mice: (**a**) blood glucose levels; (**b**) plasma insulin levels; (**c**) body weight of WT and β2-chimaerin KO (β2-KO) male mice in fasting and postprandial (PP) conditions at 2–3 months of age. Results are expressed as mean ± SEM (*n* = 10 WT, 11 β2-KO); (**d**) body weight evolution of WT and β2-KO male mice (*n* = 5–7 mice per group). Body weight was measured weekly from age 4 to 26 weeks.

To investigate the role of β2-chimaerin in regulating glucose homeostasis in vivo, we studied the effect of downregulating this protein using whole-body β2-chimaerin KO mice (hereafter β2-KO), that were generated by gene trapping [27]. Initially, we assessed the parameters of glucose homeostasis in these mice (Figure 1). Blood glucose and plasma insulin levels were comparable between β2-KO and wild-type (WT) littermates in both fasted and postprandial states (Figure 1a,b). Body weights of mice from both genotypes were also comparable in fasting and fed conditions and showed similar evolution throughout 26 weeks (Figure 1c,d).

**Figure 2:**
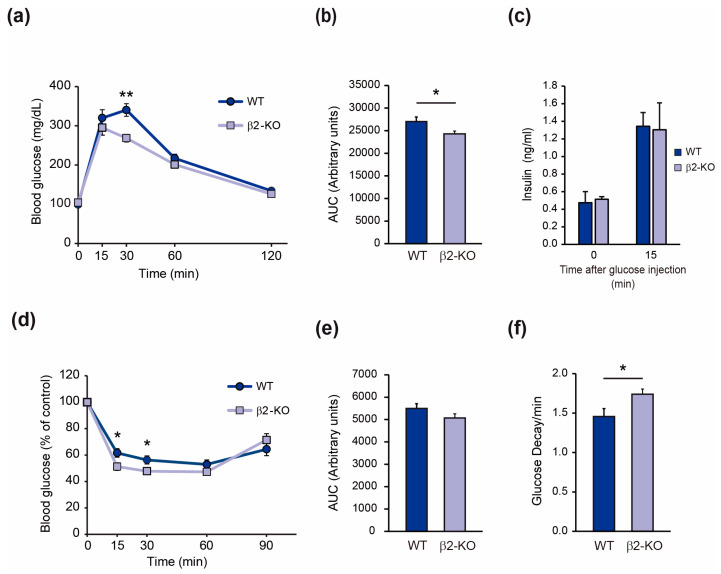
β2-chimaerin KO mice have resulted in improved glucose tolerance and increased insulin sensitivity: (**a**) Intraperitoneal glucose tolerance test (IPGTT) of male mice at 2–4 months of age (*n* = 10 WT, 11 β2-KO); (**b**) Area under the curve (AUC) of the IPGTT; (**c**) Plasma insulin levels during an IPGTT (*n* = 4–5 per group); (**d**) Intraperitoneal Insulin tolerance test (IPITT) of male mice at 2–3 months of age (*n* = 10 per group); (**e**) Area under the curve (AUC) of the IPITT; (**f**) Glucose decay after first 30 min of insulin injection during the IPITT. Results are shown as mean ± SEM. Statistical significance was assessed by two-tailed, unpaired Student’s *t*-test (* *p* ≤ 0.05, ** *p* ≤ 0.01).

We next performed an intraperitoneal glucose tolerance test (IPGTT) (Figure 2). Blood glucose levels in β2-KO mice, decreased more rapidly than in WT littermates following glucose injection (Figure 2a), with a statistically significant difference observed at 30 min. The difference in glycemic levels during the IPGTT was confirmed by the areas under the curve (AUC), which were also significantly decreased in β2-KO mice (Figure 2b). These results demonstrate that β2-KO mice exhibit improved glucose tolerance.

To determine whether this effect was due to altered insulin secretion, we measured plasma insulin levels during the glucose tolerance test. The increase in plasma insulin levels after glucose injection was similar in WT and β2-KO mice (Figure 2c), thus discarding an effect of β2-chimaerin ablation on insulin levels as the cause of the enhanced glucose tolerance observed in the β2-KO mice.

To assess the whole-body insulin sensitivity of β2-KO mice, we performed an intraperitoneal insulin tolerance test (IPITT). Glucose levels of β2-KO mice were significantly lower at 15 and 30 min after insulin injection compared to WT littermates (Figure 2d), with differences in the AUC showing a trend towards significance (*p*-value = 0.08) (Figure 2e). Glucose decay during the first 30 min of the IPITT was significantly improved in the β2-KO mice (Figure 2f). Altogether, these results are indicative of an improved glucose tolerance of β2-KO mice due to increased insulin sensitivity in peripheral tissues. Additionally, no sex-associated differences were observed in the parameters analyzed (Figure S1).

### 2.2. β2-Chimaerin Modulates Insulin Signaling in the Liver

**Figure 3:**
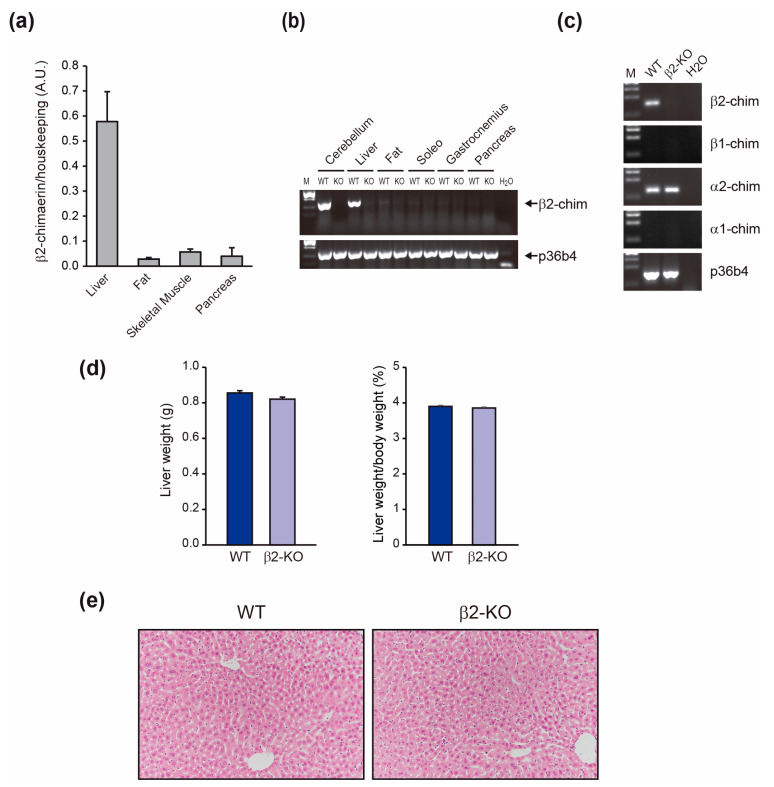
Analysis of β2-chimaerin expression in insulin-sensitive tissues: (**a**) Quantitative-RT-PCR analysis of β2-chimaerin expression in peripheral insulin-responsive tissues and pancreas from WT mice (*n* = 3). The expression of the housekeeping gene p36b4 was used for normalization. Results are expressed as mean ± SEM; (**b**) Electrophoretic analysis of the quantitative RT-PCR products in WT and β2-KO mice. Expression in cerebellum was included as a positive control. M: ΦX174 DNA/HaeIII marker; (**c**) Electrophoretic analysis of the quantitative RT-PCR products of β2-, β1-, α2- and α1-chimaerin expression in liver from WT and β2-KO mice. M: ΦX174 DNA/HaeIII marker; (**d**) Liver weight and liver/body weight of WT and β2-KO mice (*n* = 6 per group). Results are expressed as mean ± SEM; (**e**) Representative H&E staining (20×) of liver from each group.

To evaluate the contribution of β2-chimaerin to insulin-mediated responses that explained the metabolic phenotype of β2-KO mice, we first analyzed its expression in the main insulin-responsive peripheral tissues (Figure 3a). Quantitative RT-PCR analysis revealed that β2-chimaerin transcript is predominantly expressed in the liver, with only residual expression in muscle and adipose tissue. Furthermore, β2-chimaerin was barely expressed in the pancreas, consistent with the observation that β2-chimaerin ablation does not affect insulin secretion. We also confirmed the lack of expression of β2-chimaerin in the β2-KO mice tissues (Figure 3b).

To rule out compensatory expression of other chimaerin isoforms in β2-KO livers, we examined mRNA levels of the other three main isoforms (β1-, α1-chimaerin and α2-chimaerin) by quantitative RT-PCR. Neither α1- nor β1-chimaerin was detected in the liver of either WT or β2-KO mice, while α2-chimaerin was expressed at comparable levels in the mouse livers of both genotypes (2^−ΔCt^ values vs. housekeeping of 0.79 × 10^−4^ and 0.74 × 10^−4^ in WT and β2-KO, respectively) (Figure 3c). We also corroborated that liver weight and histology were similar in WT and β2-KO mice, indicating that β2-chimaerin deletion has no obvious adverse effects on the liver (Figure 3d,e).

Since β2-chimaerin was detected exclusively in the liver, we next evaluated the impact of β2-chimaerin ablation on insulin signaling in this tissue. To this end, we administrated insulin (0.75 mU/g) or saline solution by intraperitoneal injection to fasted β2-KO and control male mice. Ten minutes post-injection, mice were euthanized, and their livers were collected for analysis.

**Figure 4:**
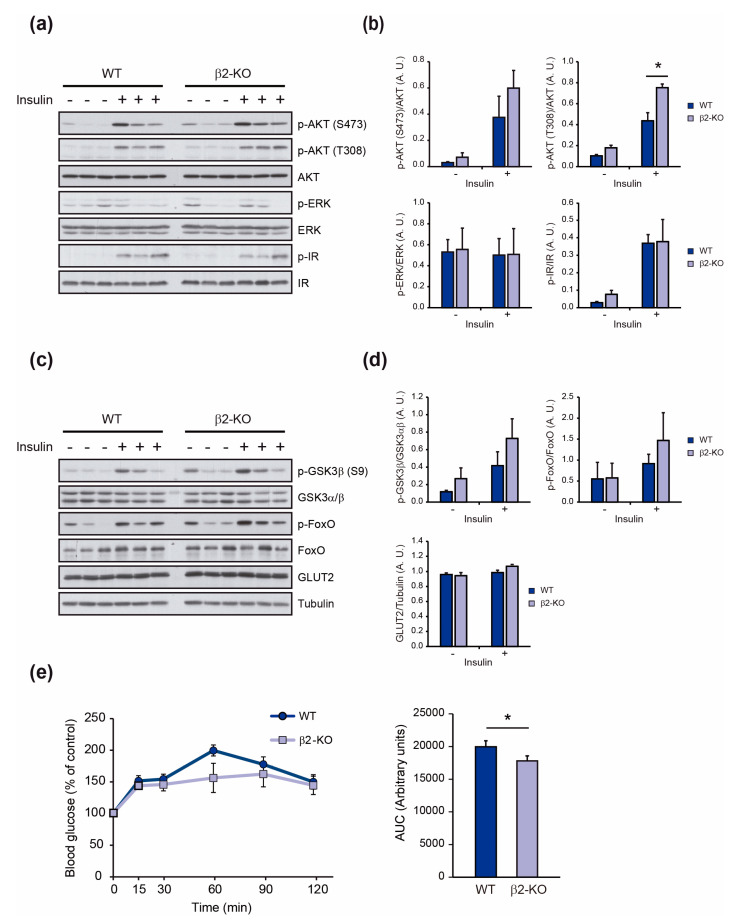
Effect of β2-chimaerin deletion on insulin signaling in the liver: (**a**) Representative immunoblot images of basal and insulin-stimulated phosphorylation of AKT, ERK and IR in liver extracts of WT and β2-KO mice. (**b**) Densitometric analysis of the phosphorylation of AKT (S473 and T308), ERK and IR. (**c**) Representative immunoblot images of basal and insulin-stimulated phosphorylation of GSK3β, FOXO, and total levels of GLUT2 in liver extracts of WT and β2-KO mice. (**d**) Densitometric analysis of p-GSK3β(S9), p-FOXO (S256), and GLUT2 levels. Results in (**b**) and (**d**) are shown as mean ± SEM (*n* = 3 per condition) and were repeated 2–3 times. (**e**) Intraperitoneal pyruvate tolerance test (IPPTT) of male mice at 2–4 months of age (*n* = 7–8 per group) and area under the curve (AUC) of the IPPTT. Results are shown as mean ± SEM. Statistical significance was assessed by unpaired, Student’s *t*-test (* *p* ≤ 0.05). A.U., arbitrary units.

Insulin signaling is initiated by its binding to the insulin receptor (IR) that triggers a cascade of phosphorylation events that orchestrates insulin’s actions. A key response in the metabolic effects of insulin is the activation of AKT, which is phosphorylated at Thr308 by PDK1 and at Ser473 by mTORC2 [28]. In the livers of β2-KO mice, AKT phosphorylation at both residues was enhanced, with Thr308 phosphorylation reaching statistical significance (Figure 4a,b). Additionally, an increase in the basal phosphorylation of AKT was observed in β2-KO livers. To confirm a liver-specific action of β2-chimaerin, we analyzed AKT phosphorylation in muscle and adipose tissues and found no differences in either basal or insulin-stimulated P-AKT (S473) levels (Figure S2).

Insulin can also activate mitogenic responses through the activation of the MAPK/ERK pathway [1]. Given that downregulation of β2-chimaerin leads to the activation of ERK downstream of tyrosine kinase receptors [29], we analyzed the phosphorylation status of this kinase. ERK was barely phosphorylated in the livers, with an inconsistent response to insulin at physiological concentrations, as reported [1], and overall phosphorylation levels were comparable between WT and β2-KO mice (Figure 4a,b). To determine whether the increased activation of AKT resulted from enhanced insulin IR activation, we assessed the insulin-mediated tyrosine phosphorylation of this receptor. β2-chimaerin deletion did not affect IR phosphorylation at Y1150/1151 following insulin stimulation, although a slight increase in basal phosphorylation was observed (Figure 4a,b).

AKT phosphorylates multiple downstream targets. Among those relevant to the control of circulating glucose levels that may explain the β2-KO phenotype are glycogen synthase kinase 3 beta (GSK3β) and the forkhead box O1 (FOXO1) transcription factor. Phosphorylation of GSK3β inhibits its activity, leading to activation of glycogen synthase (GS) and, consequently, increased glycogen synthesis [30]. Conversely, phosphorylation of FOXO1 triggers its nuclear exclusion, thereby inhibiting its role in promoting gluconeogenesis [31]. We examined AKT-mediated phosphorylation of these proteins and found that β2-KO livers exhibited enhanced phosphorylation of both GSK3β and FOXO1 compared to WTs (Figure 4c,d), although differences did not reach statistical significance (*p*- values of 0.33 and 0.41, respectively). Additionally, we evaluated the levels of the hepatic GLUT2, the major glucose transporter of hepatocytes, and found no differences between WT and β2-KO livers (Figure 4c,d).

To explore the functional impact of the altered signaling observed in β2-KO livers, we performed a pyruvate tolerance test. In line with the increased phosphorylation of FOXO1, β2-KO mice exhibited reduced gluconeogenesis, as indicated by lower glucose levels following intraperitoneal administration of pyruvate (Figure 4e).

Taken together, these data suggest that the improved glucose tolerance observed in β2-KO mice results from increased AKT phosphorylation in the liver in the absence of β2-chimaerin, which enhances downstream signaling that regulates gluconeogenesis and glycogen synthesis.

### 2.3. Insulin Activates Rac1 in the Liver Independently of β2-Chimaerin Downregulation

**Figure 5:**
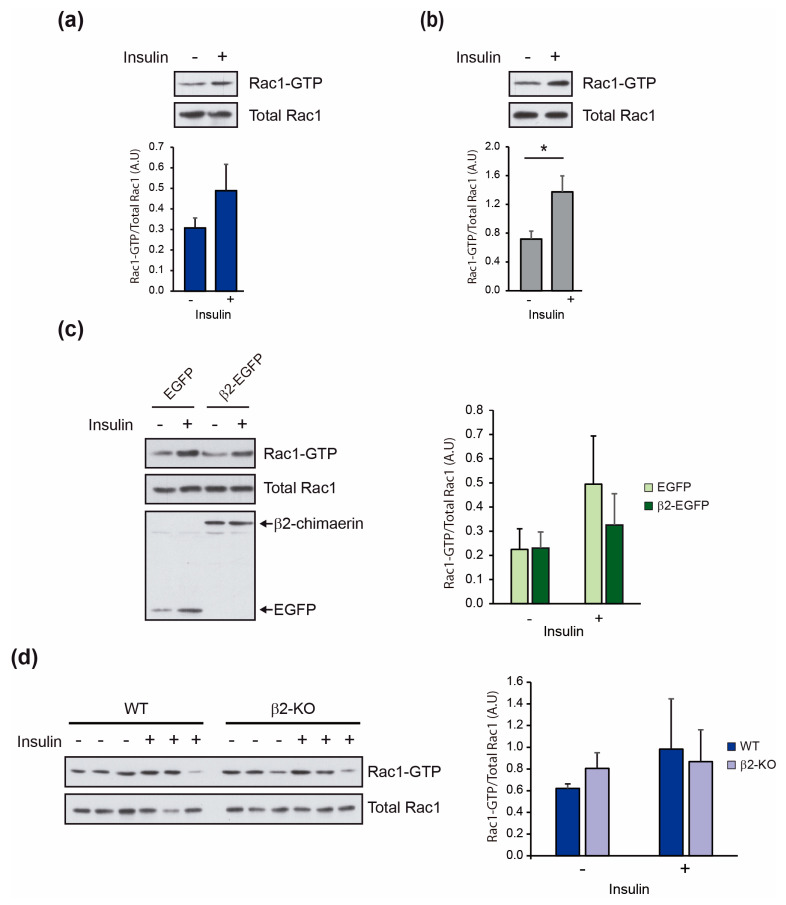
Effect of β2-chimaerin on insulin-mediated Rac1 activation in the liver: (**a**) Rac1-GTP levels in liver extracts of WT mice in fasted condition (−) or after intraperitoneal injection of insulin (0.75 mU/g) (+). (**b**) Rac1-GTP levels in HepG2 cells in serum-starved condition (−) or stimulated with insulin (100 nM) for 10 min (+). (**c**) Rac1-GTP levels in HepG2 cells transfected with EGFP or β2-chimaerin expression vectors and stimulated as in (**b**). (**d**) Rac1-GTP levels in liver extracts of WT and β2-KO mice in fasted condition (−) or after intraperitoneal injection of insulin (0.75 mU/g) (+). A representative Rac1 pull-down assay is shown in each figure. Histograms show the quantification of Rac1-GTP levels normalized to the total Rac1. Results are shown as mean ± SEM (*n* = 3–4). Rac1 pull-down assay in (**d**) was repeated 3 times. Statistical significance was assessed by two-tailed, unpaired Student’s *t*-test (* *p* ≤ 0.05). A.U., arbitrary units.

The biological functions of β2-chimaerin described thus far are associated with its ability to inactivate Rac1 [27,32,33]. Given the lack of reports on insulin’s effect on hepatic Rac1 activity, we first investigated this issue by performing a Rac1 pull-down assay on control and insulin-stimulated mouse liver samples (Figure 5a). Remarkably, endogenous Rac1-GTP levels increased by ~59% following insulin stimulation, demonstrating for the first time that insulin activates Rac1 in the liver, although this difference was not statistically significant (*p* = 0.29). To corroborate this finding, we performed the same assay in HepG2 cells and observed a statistically significant activation of Rac1 in response to insulin stimulation, consistent with the results obtained from liver extracts (Figure 5b).

Next, we investigated the specific role of β2-chimaerin in this activation. As a first approach, we ectopically expressed an EGFP-tagged β2-chimaerin in HepG2 and compared Rac1 activation to that of cells expressing EGFP as control (Figure 5c). Overexpression of β2-chimaerin partially inhibited insulin-mediated Rac1 activation, consistent with previous reports of β2-chimaerin’s inhibitory effect on Rac1 activation by EGF or heregulin [27,34,35]. We then studied the effect of β2-chimaerin downregulation on Rac1 activity in the liver. Unexpectedly, Rac1-GTP levels were similar in insulin-stimulated WT and β2-KO liver extracts, although a slight increase in basal Rac1 activation was observed in β2-KO livers. This result suggests that ablation of β2-chimaerin is not sufficient to induce altered Rac1 activation after insulin stimulation, which points to a Rac1-independent function of this GAP in the regulation of insulin signaling and/or the existence of compensatory mechanisms that maintain proper Rac1 activation.

## 3. Discussion

Rac1 is an important regulator of glucose homeostasis. Despite the liver playing a significant role in this process, whether Rac1 and/or its regulatory proteins have glucoregulatory roles in this organ is largely unknown. In this study, we show for the first time that insulin activates Rac1 in the liver and identify β2-chimaerin as a GAP protein that modulates insulin signaling in this organ.

Using a whole-body β2-chimaerin KO mice, we demonstrate that deletion of this protein results in improved glucose tolerance and enhanced insulin sensitivity. Initially, we hypothesized that this effect might stem from increased glucose transport by skeletal muscle and/or adipose tissue due to enhanced Rac1 activation, given the established role of Rac1 in glucose uptake within these tissues [9,10,14]. However, the expression pattern of β2-chimaerin in peripheral insulin-sensitive tissues revealed exclusive expression of this GAP in the liver, thereby excluding the skeletal muscle as the site of action for this protein. We therefore focused on the effect of β2-chimaerin deletion on insulin signaling in the liver and found increased activation of AKT and enhanced phosphorylation, and consequent inactivation, of its downstream targets GSK3β and FOXO1 in the β2-KO mice. The absence of significant effects on AKT phosphorylation in muscle and adipose tissue further supports a liver-specific role for β2-chimaerin.

Enhanced activation of glycogen synthesis via inhibition of GSK3β and reduced gluconeogenesis through inhibition of FOXO1-mediated gluconeogenic gene expression in β2-KO livers can explain the increased glucose-lowering action of insulin observed in these mice. This result aligns with the established role of β2-chimaerin in various cell contexts, where it restrains signaling initiated by the activation of cell surface receptors. For example, β2-chimaerin modulates signaling from semaphorins receptors in neuronal cells [32], the EGF receptor in epithelial cells [34] and the T cell receptor in lymphocytes [33]. Our data support a similar role for β2-chimaerin in hepatocytes, acting as a negative regulator of IR signaling.

We investigated the molecular mechanisms mediating β2-chimaerin’s effect, focusing on Rac1 activation since all previously described actions of β2-chimaerin are mediated through the inhibition of this GTPase [27,29,34]. We first demonstrate that insulin activates endogenous Rac1 in the liver and in the HepG2 hepatic cell line, similarly to the insulin action in skeletal muscle [11]. β2-chimaerin, when overexpressed, is capable of attenuating insulin-mediated Rac1 activation, thus positioning this protein as a regulator of Rac1 signaling activated by insulin. However, downregulation of β2-chimaerin did not result in increased levels of active Rac1, as observed in other cell models [34,36] and in vivo studies [27,29]. The discrepancy on these results may be attributed to compensatory mechanisms. We ruled out that other members of the chimaerin family compensate for the downregulation of β2-chimaerin, as their expression levels were similar in both WT and β2-KO livers. It will be important to identify other Rac1 GAPs expressed in the liver and elucidate their role in maintaining proper Rac1 activity. Notably, several Rac1 GEFs are expressed in the liver, suggesting that a fully functional Rac1 regulatory cycle operates within this organ [21].

Another plausible explanation is that β2-chimaerin modulates insulin signaling through a Rac1-independent mechanism. The increased phosphorylation of AKT observed in β2-KO livers is unlikely to be a consequence of enhanced Rac1 activation, as in most cellular contexts, including the insulin-signaling cascade in skeletal muscle, Rac1 and AKT participate in independent pathways, or Rac1 acts downstream of AKT [37,38]. Therefore, β2-chimaerin’s effect on insulin signaling may be mediated by its regulatory domains rather than its catalytic GAP domain. β2-chimaerin contains a C1 domain that binds DAG and an SH2 domain that binds phospho-tyrosine motifs [39,40]. These domains influence β2-chimaerin’s functions by regulating its subcellular localization and interactions with proteins and lipids [40]. This multi-domain structure is common in Rho regulatory proteins and there are examples of GAPs and GEFs acting as scaffolds in insulin signaling, such as TCGAP and P-REX2 [41,42]. In line with a GAP-independent function of β2-chimaerin, the phenotype of the β2-KO mice is opposite to that of the Rac1 GEF P-Rex2, which shows reduced AKT and GSK3β phosphorylation in the liver through a mechanism involving its regulatory PH domain and independent on its effect on Rac1 activity [42].

Our findings highlight the need for further research in two important issues. First, the discovery that insulin activates Rac1 in the liver suggests that active Rac1 has a yet undefined role in the hepatic insulin signaling cascade. In contrast to muscle cells and adipocytes, where insulin activates Rac1 to promote GLUT4 translocation via actin cytoskeleton rearrangements for glucose uptake [9,10,11,14], glucose transport in hepatocytes, mediated by GLUT2, is insulin-independent [43,44]. Whether Rac1’s regulation of the cytoskeleton is relevant to other insulin-mediated processes in the liver or whether Rac1 exerts regulatory roles beyond cytoskeletal control remains an open question for further investigation.

Another important issue is the role of β2-chimaerin in the development of insulin resistance and diabetes. A simultaneous reduction in β2-chimaerin and IR expression leads to a complex syndrome characterized by severe insulin resistance, diabetes, and growth deficiency in humans [24]. Therefore, it is essential to explore how β2-chimaerin influences insulin signaling in states of insulin resistance. Notably, whole-body knockout models of other regulators of small G proteins, such as Vav3 and ArhGAP21, exhibit varying metabolic phenotypes depending on dietary conditions [45,46].

In conclusion, our findings expand on previous research on the emerging roles of Rac1 and its regulators in glucose homeostasis, identifying β2-chimaerin as a novel modulator of insulin signaling in the liver.

## 4. Materials and Methods

### 4.1. Mice

Mice were housed at the Animal Research Facility of the University of Valladolid. All animal care and protocols were reviewed and approved by the Institutional Animal Care and Use Committee and the Animal Experimentation authorities of the autonomous government of Castilla and León (Spain) (experimental project approval number: 10006959), and complied with the European Community directive 2010/63/EU.

Mice containing a gene-trap insertion in the β2-chimaerin gene (*Chn2*) were obtained from Lexicon Genetics (Woodland, TX, USA) and were described before [27]. Mice were originally in a mixed C57Bl/6/129/SvEvBrd background and were backcrossed to the C57Bl/6 background for 7 generations. The colony was maintained by heterozygous mating. Genotyping was performed by PCR of mouse tail DNA according to Lexicon’s recommendations. All experiments were performed on age-matched (8–12 weeks old), cohoused littermates.

### 4.2. Mouse Metabolic Studies

Glucose tolerance, insulin sensitivity, and gluconeogenesis were assessed using the intraperitoneal glucose tolerance test (IPGTT), insulin tolerance test (IPITT) and pyruvate tolerance test (IPPTT), respectively, as previously described [47,48]. For the IPGTT, mice were fasted for 16 h and injected intraperitoneally with glucose at 2 g/kg body weight. For the IPITT, mice were injected intraperitoneally with human insulin (Humulin; Eli Lilly, Indianapolis, USA) at a dose of 0.75 U/kg body weight. For the IPPTT, after a 16 h fast, mice were injected intraperitoneally with sodium pyruvate (2 g/kg body weight). For all these tests, glycemia was monitored before and at various time points post-injection from tail blood glucose using a Contour XT glucometer (Ascensia Diabetes Care). The area under the curve was calculated by the trapezoidal method [49]. Glucose decay was calculated as described [50]. Plasma insulin was determined using an ultrasensitive mouse insulin kit (Crystal Chem Inc., Downers Grove, IL, USA) according to the manufacturer’s instructions.

### 4.3. In Vivo Insulin Signaling

Mice were fasted overnight and injected with 0.75 U/kg of body weight of insulin (Humulin R; Eli Lilly) or saline (control group). After 10 min, mice were sacrificed by cervical dislocation under sedation. Tissues were collected, immediately frozen in liquid nitrogen, and stored at −80 °C until processing for protein analysis and Rac1 activation assay. A piece of liver was also fixed with 4% formalin for 24 h for histological analysis.

### 4.4. Quantitative Real-Time PCR

Quantitative RT-PCR analysis was performed with the QuantiTect SYBR Green RT-PCR Kit (Qiagen, Hilden, Germany), using 150 ng of total RNA from mice tissues isolated with TRIzol reagent (Invitrogen, Carlsbad, CA, USA). The p36b4 gene was used as housekeeping. Primers were as follows: β2-chimaerin forward 5′-AGGTGGAAAACAGACCAAAA-3′, reverse 5′-CCCAAACCTCAGTGCCAGCG-3′; β1-chimaerin forward 5′-TCAAGCTCTTTGCCTGTTC-3′, reverse 5′-CATAAAATTGGCACAGTATTC-3′; α2-chimaerin forward 5′-ATACAGATGAATATAGACCTCC-3′, reverse 5′- CCATGATACTCTCTTCCATAA-3′; α1-chimaerin forward 5′-CCTCTGAAACTCTTTGCTTA-3′, reverse 5′-GGCACAGTATTCACACCAG-3′; p36b4 forward 5′-GTGTTTGACAACGGCAGCATT-3′, reverse 5′-TTGATGATGGAGTGTGGCACC-3′. Quantitative RT-PCR was performed on a Light Cycler^®^ 480 Real-Time PCR System (Roche Diagnostics). Expression of chimaerin isoforms was normalized to that of the housekeeping gene expression using a standard 2^−ΔCT^ method.

### 4.5. Western Blot Assay

Tissues (20 mg) were homogenized in a lysis buffer containing 20 mM Tris-HCl (pH 7.4), 150 mM NaCl, 0.5 mM EDTA, 1 mM Na_3_VO_4_, 1 mM NaF, 1 mM DTT and a mixture of protease inhibitors (Cømplete, Roche Molecular Biochemicals, Mannheim, Germany), using a Precellys^®^ 24 tissue homogenizer (Bertin Instruments, Berlin, Germany). After homogenization, Triton X-100 was added to a final 1% concentration. Tissue lysates were centrifuged at 12,000× *g* for 10 min at 4 °C to remove cell debris, and protein content was quantified using the Bradford method. Equivalent amounts of protein were resolved by SDS-PAGE and processed by immunoblotting analysis [51]. The following primary antibodies were used: antibodies against p-AKT (Ser473) (#4060), p-AKT (Thr308) (#13038), AKT (#9272), p-ERK (Thr202/Tyr204) (#9101), ERK (#9102), p-IR (Tyr1150/1151) (#3024), p-GSK-3β (Ser9) (#5558), p-FoxO1 (Ser256) (#9461) and FoxO (#2880) were from Cell Signaling Technology; IR (#A303-712A-T) from Bethyl, GSK-3 α/β (#VMA00342) from BioRad, GLUT2 (# 07-1402-I) from Proteintech, Tubulin (#CP-06) from Oncogene, and GFP (#MMs-118P) from Covance. Immunoblot–derived signals were quantified using Quantity One-1D.

### 4.6. Cell Culture and Transfection

HepG2 cells were kindly provided by Dr. Jose Juan García Marín (University of Salamanca, Salamanca, Spain). Cells were grown at 37 °C and 5% CO_2_ in DMEM (1 g/L D-glucose) supplemented with 10% fetal bovine serum (FBS), 1% L-glutamine and 100 units/mL penicillin and streptomycin (Gibco, New York, USA). Transfection with the expression vectors for EGFP-tagged β2-chimaerin (pEGFP-C3-β2) [39] or EGFP was performed by using X-tremeGENE HP (Roche, Mannheim, Germany), according to the manufacturer protocol.

### 4.7. Rac1 Activation Assay

Rac1-GTP levels in HepG2 and liver extracts were assessed by pull-down assay with a GST fusion protein containing the Rac1 binding domain of PAK1 (GST-PBD) as described previously [27]. Briefly, HepG2 cell lines were seeded in six-well plates, serum-starved overnight and then stimulated with insulin (100 nM) for 10 min. After stimulation cells were lysed in 400 μL of lysis buffer containing 20 mM Tris-HCl (pH 7.5), 150 mM NaCl, 5 mM MgCl_2_, 1% Triton X-100, 1 mM DTT, 1 mM Na_3_VO_4_, 1 mM NaF, protease inhibitors (Cømplete, Roche Diagnostics), and 10 μg/sample of GST-PBD. Liver samples (40 mg) were homogenized in the same lysis buffer (1:40 *w*/*v*) without detergent, using a Precellys^®^ 24 tissue homogenizer (Bertin Instruments, Berlin, Germany), and then Triton X-100 was added to a final 1% (*v*/*v*) concentration. Cleared cell and liver lysates were incubated with glutathione-Sepharose beads (GE Healthcare, Uppsala, Sweden) for 1 h at 4 °C. After incubation, samples were extensively washed, boiled in SDS-PAGE sample buffer and separated by electrophoresis. Bound Rac1 proteins were detected by immunoblotting using anti-Rac antibody (610651, BD Transduction Laboratories, Franklin Lakes, NJ, USA). Rac1-GTP levels were quantitated using the Quantity One 1D image analysis 4.5 software (Bio-Rad, Hercules, CA, USA) and were normalized to total Rac1.

### 4.8. Statistical Analyses

All statistical analyses were performed using GraphPad Prism 8.0 (GraphPad Software, Inc., Boston, MA, USA). Data are presented as mean ± SEM. Comparisons between the two groups were carried out using the two-tailed unpaired Student’s *t*-test. Differences were considered significant at *p* < 0.05.

## Data Availability

The data presented in this study are available on request from the corresponding author.

## Supplementary Materials

The following supporting information can be downloaded at: https://www.mdpi.com/article/10.3390/molecules29225301/s1, Figure S1: Metabolic features of β2-chimaerin KO female mice. Figure S2. Effect of β2-chimaerin deletion on AKT phosphorylation in adipose tissue and muscle.

## References

[1] Bedinger D.H., Adams S.H. (2015). Metabolic, anabolic, and mitogenic insulin responses: A tissue-specific perspective for insulin receptor activators. Mol. Cell. Endocrinol..

[2] Møller L.L.V., Klip A., Sylow L. (2019). Rho GTPases-Emerging Regulators of Glucose Homeostasis and Metabolic Health. Cells.

[3] Satoh T. (2014). Rho GTPases in insulin-stimulated glucose uptake. Small GTPases.

[4] Thurmond D.C., Gonelle-Gispert C., Furukawa M., Halban P.A., Pessin J.E. (2003). Glucose-stimulated insulin secretion is coupled to the interaction of actin with the t-SNARE (target membrane soluble N-ethylmaleimide-sensitive factor attachment protein receptor protein) complex. Mol. Endocrinol..

[5] Asahara S., Shibutani Y., Teruyama K., Inoue H.Y., Kawada Y., Etoh H., Matsuda T., Kimura-Koyanagi M., Hashimoto N., Sakahara M. (2013). Ras-related C3 botulinum toxin substrate 1 (RAC1) regulates glucose-stimulated insulin secretion via modulation of F-actin. Diabetologia.

[6] Tong P., Khayat Z.A., Huang C., Patel N., Ueyama A., Klip A. (2001). Insulin-induced cortical actin remodeling promotes GLUT4 insertion at muscle cell membrane ruffles. J. Clin. Investig..

[7] Kanzaki M., Pessin J.E. (2001). Insulin-stimulated GLUT4 translocation in adipocytes is dependent upon cortical actin remodeling. J. Biol. Chem..

[8] Tsakiridis T., Vranic M., Klip A. (1994). Disassembly of the actin network inhibits insulin-dependent stimulation of glucose transport and prevents recruitment of glucose transporters to the plasma membrane. J. Biol. Chem..

[9] Sylow L., Jensen T.E., Kleinert M., Højlund K., Kiens B., Wojtaszewski J., Prats C., Schjerling P., Richter E.A. (2013). Rac1 signaling is required for insulin-stimulated glucose uptake and is dysregulated in insulin-resistant murine and human skeletal muscle. Diabetes.

[10] Ueda S., Kitazawa S., Ishida K., Nishikawa Y., Matsui M., Matsumoto H., Aoki T., Nozaki S., Takeda T., Tamori Y. (2010). Crucial role of the small GTPase Rac1 in insulin-stimulated translocation of glucose transporter 4 to the mouse skeletal muscle sarcolemma. FASEB J..

[11] JeBailey L., Rudich A., Huang X., Di Ciano-Oliveira C., Kapus A., Klip A. (2004). Skeletal muscle cells and adipocytes differ in their reliance on TC10 and Rac for insulin-induced actin remodeling. Mol. Endocrinol..

[12] Usui I., Imamura T., Huang J., Satoh H., Olefsky J.M. (2003). Cdc42 is a Rho GTPase family member that can mediate insulin signaling to glucose transport in 3T3-L1 adipocytes. J. Biol. Chem..

[13] Karnam P., Standaert M.L., Galloway L., Farese R.V. (1997). Activation and translocation of Rho (and ADP ribosylation factor) by insulin in rat adipocytes. Apparent involvement of phosphatidylinositol 3-kinase. J. Biol. Chem..

[14] Takenaka N., Nakao M., Matsui S., Satoh T. (2019). A Crucial Role for the Small GTPase Rac1 Downstream of the Protein Kinase Akt2 in Insulin Signaling that Regulates Glucose Uptake in Mouse Adipocytes. Int. J. Mol. Sci..

[15] Syed I., Kyathanahalli C.N., Jayaram B., Govind S., Rhodes C.J., Kowluru R.A., Kowluru A. (2011). Increased phagocyte-like NADPH oxidase and ROS generation in type 2 diabetic ZDF rat and human islets: Role of Rac1-JNK1/2 signaling pathway in mitochondrial dysregulation in the diabetic islet. Diabetes.

[16] Zhou S., Yu D., Ning S., Zhang H., Jiang L., He L., Li M., Sun M. (2015). Augmented Rac1 Expression and Activity are Associated with Oxidative Stress and Decline of β Cell Function in Obesity. Cell Physiol. Biochem..

[17] Sylow L., Kleinert M., Pehmøller C., Prats C., Chiu T.T., Klip A., Richter E.A., Jensen T.E. (2014). Akt and Rac1 signaling are jointly required for insulin-stimulated glucose uptake in skeletal muscle and downregulated in insulin resistance. Cell Signal..

[18] Jaffe A.B., Hall A. (2005). Rho GTPases: Biochemistry and biology. Annu. Rev. Cell Dev. Biol..

[19] Cherfils J., Zeghouf M. (2013). Regulation of small GTPases by GEFs, GAPs, and GDIs. Physiol. Rev..

[20] Hall A. (2012). Rho family GTPases. Biochem. Soc. Trans..

[21] Machin P.A., Tsonou E., Hornigold D.C., Welch H.C.E. (2021). Rho Family GTPases and Rho GEFs in Glucose Homeostasis. Cells.

[22] Møller L.L.V., Ali M.S., Davey J., Raun S.H., Andersen N.R., Long J.Z., Qian H., Jeppesen J.F., Henriquez-Olguin C., Frank E. (2023). The Rho guanine dissociation inhibitor α inhibits skeletal muscle Rac1 activity and insulin action. Proc. Natl. Acad. Sci. USA.

[23] Caloca M.J., Wang H., Kazanietz M.G. (2003). Characterization of the Rac-GAP (Rac-GTPase-activating protein) activity of beta2-chimaerin, a ‘non-protein kinase C’ phorbol ester receptor. Biochem. J..

[24] Suliman S.G., Stanik J., McCulloch L.J., Wilson N., Edghill E.L., Misovicova N., Gasperikova D., Sandrikova V., Elliott K.S., Barak L. (2009). Severe insulin resistance and intrauterine growth deficiency associated with haploinsufficiency for INSR and CHN2: New insights into synergistic pathways involved in growth and metabolism. Diabetes.

[25] Keaton J.M., Hellwege J.N., Ng M.C., Palmer N.D., Pankow J.S., Fornage M., Wilson J.G., Correa A., Rasmussen-Torvik L.J., Rotter J.I. (2016). Genome-Wide Interaction with Insulin Secretion Loci Reveals Novel Loci for Type 2 Diabetes in African Americans. PLoS ONE.

[26] Hu C., Zhang R., Yu W., Wang J., Wang C., Pang C., Ma X., Bao Y., Xiang K., Jia W. (2011). CPVL/CHN2 genetic variant is associated with diabetic retinopathy in Chinese type 2 diabetic patients. Diabetes.

[27] Casado-Medrano V., Barrio-Real L., García-Rostán G., Baumann M., Rocks O., Caloca M.J. (2016). A new role of the Rac-GAP β2-chimaerin in cell adhesion reveals opposite functions in breast cancer initiation and tumor progression. Oncotarget.

[28] Petersen M.C., Shulman G.I. (2018). Mechanisms of Insulin Action and Insulin Resistance. Physiol. Rev..

[29] Bruinsma S.P., Cagan R.L., Baranski T.J. (2007). Chimaerin and Rac regulate cell number, adherens junctions, and ERK MAP kinase signaling in the Drosophila eye. Proc. Natl. Acad. Sci. USA.

[30] Cross D.A., Alessi D.R., Cohen P., Andjelkovich M., Hemmings B.A. (1995). Inhibition of glycogen synthase kinase-3 by insulin mediated by protein kinase B. Nature.

[31] Gross D.N., Wan M., Birnbaum M.J. (2009). The role of FOXO in the regulation of metabolism. Curr. Diab. Rep..

[32] Riccomagno M.M., Hurtado A., Wang H., Macopson J.G., Griner E.M., Betz A., Brose N., Kazanietz M.G., Kolodkin A.L. (2012). The RacGAP β2-Chimaerin selectively mediates axonal pruning in the hippocampus. Cell.

[33] Caloca M.J., Delgado P., Alarcón B., Bustelo X.R. (2008). Role of chimaerins, a group of Rac-specific GTPase activating proteins, in T-cell receptor signaling. Cell Signal..

[34] Wang H., Yang C., Leskow F.C., Sun J., Canagarajah B., Hurley J.H., Kazanietz M.G. (2006). Phospholipase Cgamma/diacylglycerol-dependent activation of beta2-chimaerin restricts EGF-induced Rac signaling. EMBO J..

[35] Yasuda S., Kai M., Imai S., Kanoh H., Sakane F. (2007). Diacylglycerol kinase gamma interacts with and activates beta2-chimaerin, a Rac-specific GAP, in response to epidermal growth factor. FEBS Lett..

[36] Yagi S., Matsuda M., Kiyokawa E. (2012). Suppression of Rac1 activity at the apical membrane of MDCK cells is essential for cyst structure maintenance. EMBO Rep..

[37] Raun S.H., Ali M., Kjøbsted R., Møller L.L.V., Federspiel M.A., Richter E.A., Jensen T.E., Sylow L. (2018). Rac1 muscle knockout exacerbates the detrimental effect of high-fat diet on insulin-stimulated muscle glucose uptake independently of Akt. J. Physiol..

[38] Takenaka N., Izawa R., Wu J., Kitagawa K., Nihata Y., Hosooka T., Noguchi T., Ogawa W., Aiba A., Satoh T. (2014). A critical role of the small GTPase Rac1 in Akt2-mediated GLUT4 translocation in mouse skeletal muscle. FEBS J..

[39] Caloca M.J., Garcia-Bermejo M.L., Blumberg P.M., Lewin N.E., Kremmer E., Mischak H., Wang S., Nacro K., Bienfait B., Marquez V.E. (1999). beta2-chimaerin is a novel target for diacylglycerol: Binding properties and changes in subcellular localization mediated by ligand binding to its C1 domain. Proc. Natl. Acad. Sci. USA.

[40] Yang C., Kazanietz M.G. (2007). Chimaerins: GAPs that bridge diacylglycerol signalling and the small G-protein Rac. Biochem. J..

[41] Chiang S.H., Hwang J., Legendre M., Zhang M., Kimura A., Saltiel A.R. (2003). TCGAP, a multidomain Rho GTPase-activating protein involved in insulin-stimulated glucose transport. EMBO J..

[42] Hodakoski C., Hopkins B.D., Barrows D., Mense S.M., Keniry M., Anderson K.E., Kern P.A., Hawkins P.T., Stephens L.R., Parsons R. (2014). Regulation of PTEN inhibition by the pleckstrin homology domain of P-REX2 during insulin signaling and glucose homeostasis. Proc. Natl. Acad. Sci. USA.

[43] Hah J., Jo I., Chakrabarti R., Jung C.Y. (1992). Demonstration of an insulin-insensitive storage pool of glucose transporters in rat hepatocytes and HepG2 cells. J. Cell. Physiol..

[44] Williams T.F., Exton J.H., Park C.R., Regen D.M. (1968). Stereospecific transport of glucose in the perfused rat liver. Am. J. Physiol..

[45] Soares G.M., Zangerolamo L., Azevedo E.G., Costa-Júnior J.M., Carneiro E.M., Saad S.T., Boschero A.C., Barbosa-Sampaio H.C. (2018). Whole body ARHGAP21 reduction improves glucose homeostasis in high-fat diet obese mice. J. Cell. Physiol..

[46] Menacho-Márquez M., Nogueiras R., Fabbiano S., Sauzeau V., Al-Massadi O., Diéguez C., Bustelo X.R. (2013). Chronic sympathoexcitation through loss of Vav3, a Rac1 activator, results in divergent effects on metabolic syndrome and obesity depending on diet. Cell Metab..

[47] Sanchez-Encinales V., Cozar-Castellano I., Garcia-Ocaña A., Perdomo G. (2015). Targeted delivery of HGF to the skeletal muscle improves glucose homeostasis in diet-induced obese mice. J. Physiol. Biochem..

[48] Cózar-Castellano I., Perdomo G. (2020). Assessment of Insulin Tolerance In Vivo in Mice. Methods Mol. Biol..

[49] Matthews J.N., Altman D.G., Campbell M.J., Royston P. (1990). Analysis of serial measurements in medical research. BMJ.

[50] Villa-Pérez P., Cueto M., Díaz-Marrero A.R., Lobatón C.D., Moreno A., Perdomo G., Cózar-Castellano I. (2017). Leptolide Improves Insulin Resistance in Diet-Induced Obese Mice. Mar. Drugs.

[51] Caloca M.J., Zugaza J.L., Bustelo X.R. (2008). Mechanistic analysis of the amplification and diversification events induced by Vav proteins in B-lymphocytes. J. Biol. Chem..

