# Supplementary material for: β2-Chimaerin, a GTPase-Activating Protein for Rac1, Is a Novel Regulator of Hepatic Insulin Signaling and Glucose Metabolism
Source: Molecules. 2024 Nov 9;29(22):5301. doi: 10.3390/molecules29225301 (PMC11597029; doi:10.3390/molecules29225301)
Supplement: Supplementary file 1 [file molecules-29-05301-s001.zip › molecules-3278776-supplementary.pdf]

β2-chimaerin, a GAP for Rac1, is a novel regulator of Hepatic Insulin Signaling and Glucose Metabolism

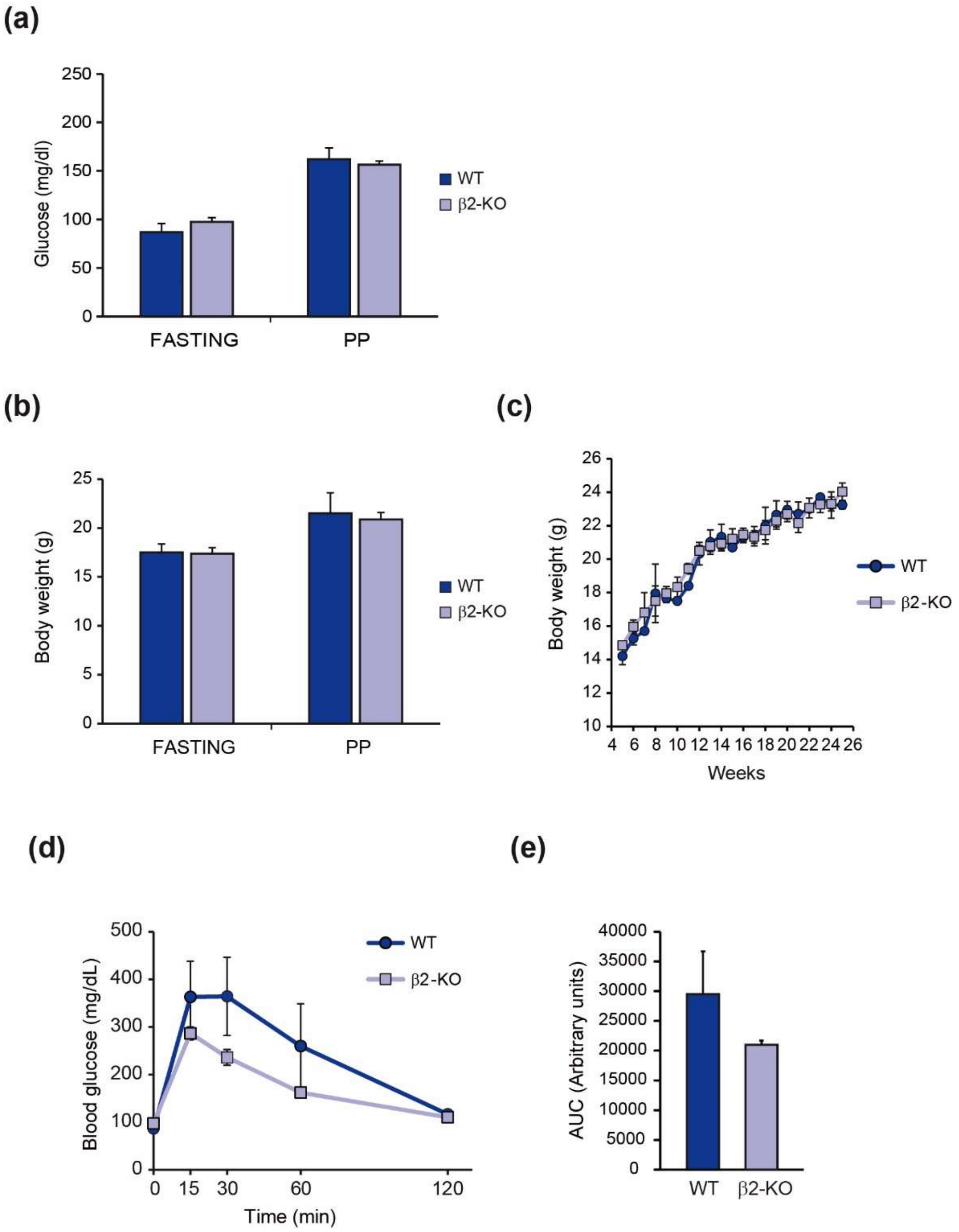

Figure S1. Metabolic features of β2-chimaerin KO female mice: **(a)** Blood glucose levels and **(b)** Body weight of WT and β2-chimaerin KO (β2-KO) female mice in fasting and postprandial (PP) conditions at 2-3 months of age (n=4 WT, 8 β2-KO); **(c)** Body weight evolution of WT and β2-KO female mice (n= 4 mice per genotype). Body weight was measured weekly from age 4-26 weeks; **(d)** Intraperitoneal glucose tolerance test (IPGTT) of female mice at 2-3 months of age (n=4 WT, 8 β2-KO); **(e)** Area under the curve (AUC) of the IPGTT. Results are expressed as mean ± SEM. Statistical significance was assessed by two-tailed, unpaired Student's t test.

(a)

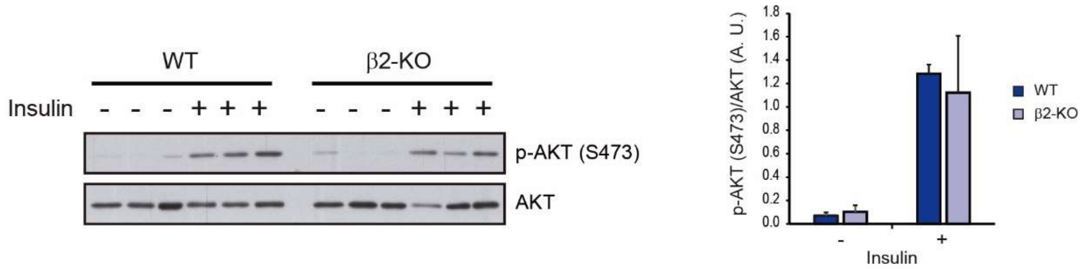

(b)

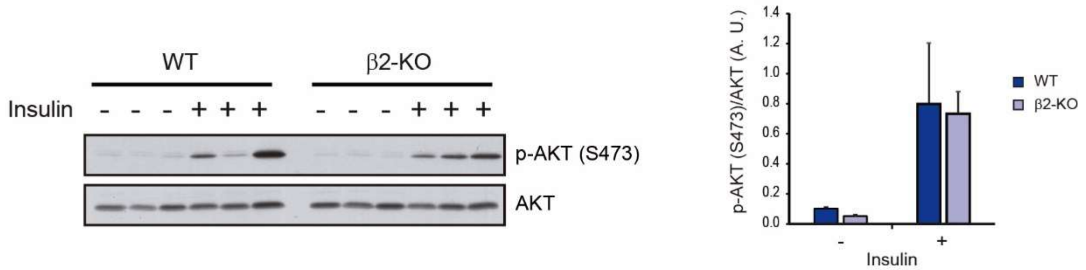

Figure S2. Effect of β2-chimaerin deletion on AKT phosphorylation in adipose tissue and muscle. Representative immunoblot images of basal and insulin-stimulated phosphorylation of AKT (S473) in (a) adipose tissue or (b) skeletal muscle (gastrocnemius) extracts from WT and β2-KO mice. Histograms display densitometric analysis of AKT phosphorylation levels. Data are presented as mean ± SEM. Statistical significance was assessed by unpaired, Student's t test (p= 0.77 and p= 0.89 for insulin-stimulated p-AKT in WT vs. β2-KO adipose tissue and muscle, respectively). A.U, arbitrary units.
